# Clinical analysis of 163 pediatric patients with infectious mononucleosis: a single‐center retrospective analysis

**DOI:** 10.1002/iid3.70020

**Published:** 2024-09-16

**Authors:** Yan Li, Kun Wang

**Affiliations:** ^1^ Department of Infectious Diseases, Children's Hospital Zhejiang University School of Medicine National Clinical Research Center for Child Health.3333 Binsheng Road, Binjiang District Hangzhou China; ^2^ Department of Infectious Disease Children's Hospital of Soochow University No.92 Zhongnan Street Suzhou China

**Keywords:** child, Epstein‐Barr Virus Infections, lactate dehydrogenases, platelet count, Retrospective Studies

## Abstract

**Objective:**

This study aims to enhance the management of Epstein‐Barr Virus (EBV) infections by analyzing the correlation between laboratory indicators and clinical manifestations in children, thereby proposing more precise diagnostic and treatment strategies.

**Methods:**

In this retrospective study included 163 pediatric patients with EBV infections treated at the Children's Hospital of Soochow University from December 2017 to December 2019. Data collected through retrospective analysis included gender, age, clinical symptoms, signs, liver function tests, T‐cell subset distribution, EBV‐DNA copy numbers in plasma, and treatment outcomes. Patients were grouped based on EBV‐DNA copy numbers in plasma and hospital stay duration to compare clinical indicators across different groups.

**Results:**

The dichotomous results of EBV‐DNA copy numbers in plasma showed that the two groups of children were significantly different in the number of days of fever (*p* = .0022), platelet count (*p* = .0212), ALT (*p* = .001), immunoglobulin IgM (*p* = .0039), IgG (*p* = .0195), TBiL (*p* = .025), LDH (*p* = 0.0001), and length of hospital stay (*p* < .001) were significantly different, indicating that EBV‐DNA copy numbers in plasma may be correlated with these characteristic variables. The dichotomous results of the length of hospital stay showed that the two groups were significantly increased in tonsil enlargement (*p* = .0024), platelet count (*p* = .0059), LDH (*p* = .0394), and ferritin (*p* = .0106) and EBV‐DNA copy numbers in plasma (*p* = 0.0361) were significantly different, This suggests a potential correlation between EBV‐DNA copy numbers in plasma and these clinical indicators.

**Conclusion:**

Variations in platelet counts and lactate dehydrogenase (LDH) levels in children with EBV infections may serve as indicators of clinical outcomes.

## 1 INTRODUCTION

The Epstein‐Barr Virus (EBV), also known as Human Herpesvirus 4, is a ubiquitous double‐stranded DNA virus found in approximately 95% of the global population^1^ and a significant pathogen in children.^2^ In developed regions like Europe and the USA, primary infections typically occur in late adolescence.^3^ However, in developing regions, infections often occur earlier,^4^ possibly due to differences in socioeconomic status. Improved economic conditions and higher education levels in coastal areas of China may have led to changes in the epidemiological features of EBV infections, necessitating additional research. EBV infections are common viral infections in children, presenting various symptoms.^5^ Primary EBV infections during childhood are often asymptomatic or atypical,^6^ which could lead to misdiagnoses or missed diagnoses.

Approximately 50% of children under 6 years old with primary EBV infection present with infectious mononucleosis (IM). Epidemiological data from China indicate that the seropositive rate for EBV infection among children aged 3 to 5 years is between 80.7% and 100.0%, reaching 100.0% by the age of 10. IM is essentially a self‐limiting lymphoproliferative disorder with a generally good prognosis, and a mortality rate of only 1–2%. Chronic active EBV infection (CAEBV) is a rare manifestation of EBV in children, characterized by recurrent or persistent IM‐like symptoms lasting for several months. CAEBV has a poor prognosis, with a mortality rate of up to 43%, often affecting multiple systems, including the hematological, digestive, respiratory, nervous, and cardiovascular systems.^7^ In rare cases, EBV‐infected children may develop EBV‐associated hemophagocytic lymphohistiocytosis (EBV‐HLH), a rapidly progressive and often fatal condition if not promptly diagnosed and treated, with a mortality rate of 30–40%. The primary EBV infection is associated with systemic interferon (IFN) responses.^8^ EBV‐mediated activation of plasmacytoid dendritic cell (pDC) TLR 9 can lead to the secretion of IFN‐α, a key factor in controlling EBV infection.^9^ Therefore, the innate immune response plays a crucial role in the body's defense against EBV. Infectious mononucleosis is the most common disease caused by EBV infection in children.

Moreover, there are few large‐sample studies on primary and reactivated EBV infections in children, and the mechanisms of asymptomatic EBV infections in children are not well understood.^10^ This study aims to further explore the clinical and laboratory characteristics of EBV infections in children to fill current research gaps.

In this study, we selected 163 children with EBV infections treated in our hospital, reviewed their clinical data, and grouped them by EBV copy numbers and hospital stay durations to compare clinical indicators across groups, thereby clarifying the correlation between laboratory and clinical indicators in children with EBV infections to improve diagnosis and treatment.

## 2 MATERIALS AND METHODS

### 2.1 Study subjects

We collected data from 163 children with EBV infections who were hospitalized for treatment at the Children's Hospital of Soochow University from December 2017 to December 2019.

### 2.2 Inclusion criteria

1) Aged ≤18 years;
2) Met the diagnostic criteria for EBV infection;
3) Only first‐time clinical data from our hospital were collected;
4) Complete clinical data available;
5) Excluded co‐infections, malignant tumors, congenital malformations, severe organ dysfunction;
6) Severe complications or had received EBV treatment were excluded.

### 2.3 Diagnostic criteria

1) Evidence of EBV infection included one of the following:

① Serological antibody detection of capsid antigen (CA) IgG and IgM antibodies positive, and CA‐IgG showing low affinity indicating primary acute or active infection;

② Molecular biology methods detecting EBV positivity in blood, bone marrow, lymph nodes, or other affected tissues.

2) Infectious mononucleosis (IM) is diagnosed based on the criteria outlined in “Zhu Futang Practical of Pediatrics”.

### 2.4 Clinical data collectionh

This study collected the following data upon hospital admission: age, gender, clinical manifestations, complete blood count results, biochemical panel (including liver function indices), humoral immunity, cellular immune function, and final outcomes.

### 2.5 Analysis

1) Statistical analysis was conducted on the gender, age, clinical manifestations, laboratory results, diagnosis, and outcomes of 163 children with EBV infections;
2) Divide the children into two groups based on EBV levels in the plasma: those with EBV‐DNA copy numbers in plasma (×10^2^) > 38.9 and those with levels ≤38.9, and compare the clinical manifestations and laboratory results between these groups.
3) Further categorize the 163 children based on the number of hospital stay days into two groups: those with more than 9 days and those with 9 days or fewer, and compare the clinical and laboratory results between these groups.

### 2.6 Statistical methods

Data analysis was performed using the R software package. Categorical variables were analyzed using the chi‐squared test with dplyr, tidyr, and victim packages. Continuous variables were analyzed using t‐tests or non‐parametric tests with dplyr, reshape, and restatix packages. A *p*‐value < .05 indicated statistical significance, and *p*‐value < .01 indicated a significant difference.

### 2.7 Ethics committee approval

This study was approved by Children's Hospital of Soochow University ethics committee and followed relevant ethical guidelines. Due to the retrospective nature of the study, patient consent was not required.

## 3 RESULTS

### 3.1 Basic patient information

This study evaluated 163 pediatric patients diagnosed with EBV infections. Detailed demographic and baseline clinical characteristics are summarized in Table 1, which provides insights into the epidemiological features of EBV in this cohort.

**Table 1:** Basic information of children.

Characteristic Variable	Variable
Sex (Male/Female)	91/72
Age (years)	4.33 (2.67–6.62)
Fever (No/Yes)	7/156(4.29%/95.71%)
Peak Fever (°C)	39.0 (38.6–39.5)
Fever Days	6 (4–10)
Tonsil Enlargement (No/I/II)	15/130/18(9.21%/79.75%/11.04%)
Cervical Lymphadenopathy (No/Yes)	14/149(8.59%/91.41%)
Hepatomegaly (No/Yes)	80/83(49.08%/50.92%)
Splenomegaly (No/Yes)	84/79(51.53%/48.47%)
Eyelid Edema (No/Yes)	89/74(54.60%/45.40%)
Rash (No/Yes)	148/15(90.79%/9.21%)
White Blood Cells (×10^9^/L)	12.68 (10.15–18.38)
Neutrophil%	23.90 (16.55–30.80)
Lymphocyte%	65.70 (58.85–74.25)
Absolute Lymphocyte Count (×10^9^/L)	8.82 (6.01–12.470)
Platelets (×10^9^/L)	211 (168–252.5)
Hemoglobin (g/L)	119 (113–124)
C‐reactive Protein (mg/L)	5.94 (2.67–13.21)
ALT (U/L)	47.5 (21.6–115)
AST (U/L)	51.2 (36.95–80.15)
lgM	1.69 (1.350–2.3)
lgG	12.07 (9.49–14.21)
lgA	1.62 (1.15–2.16)
TBiL (umol/L)	4.7 (3.9–6.0)
LDH (U/L)	549 (460.65–637.05)
CD3+ Absolute Count(×10^9^/L)	7.4 (4.84–10.19)
CD4+ Absolute Count(×10^9^/L)	1.35 (0.88–2.12)
CD8+ Absolute Count(×10^9^/L)	5.18 (3.32–7.32)
CD4 + /CD8+	0.30 (0.20–0.45)
CD19+ Absolute Count(×10^9^/L)	0.51 (0.29–0.80)
CD(16 + 56)+ Absolute Count(×10^9^/L)	0.7 (0.44–1.24)
CD19 + CD23+ Absolute Count(×10^9^/L)	0.24 (0.13–0.44)
Blood Smear Neutrophil%	24 (16‐34)
Blood Smear Lymphocyte%	64 (56.5‐72.0)
Blood Smear Atypical Lymphocyte Ratio%	4 (0‐6)
Ferritin (ug/L)	110.3 (73.1‐163.9)
EBV‐DNA copy numbers in plasma (×10^2^ copies/ml)	38.9 (16.4‐122.5)
Hospital Days	9 (7‐12)

ALT: Alanine aminotransferase; AST: Aspartate amino transferase; TBiL: Total Bilirubin; LDH: Lactate dehydrogenase; IgM: Immunoglobulin M; IgG: Immunoglobulin G; IgA: Immunoglobulin A.

#### 3.1.1 Clinical presentation and epidemiological insights

Fever: The majority of patients (95.71%) presented with fever, exhibiting a peak median temperature of 39.0°C. The median duration of fever was 6 days, indicative of acute infection phases in the majority of cases.

Organomegaly: Approximately half of the cohort exhibited hepatomegaly (50.92%) and splenomegaly (48.47%), suggesting significant systemic involvement.

#### 3.1.2 Hematological and biochemical profiles

Complete Blood Count: The analysis included white blood cells, neutrophil percentage, lymphocyte percentage, absolute lymphocyte count, platelets, hemoglobin, and C‐reactive protein. The median white blood cell count was 12.68×10^9^/L, with lymphocytes predominating (median lymphocyte percentage: 65.70%), consistent with a typical response to viral infection.

Platelets: The median platelet count was 211×10^9^/L, within the normal range but leaning towards the lower limit, suggesting a potential trend towards mild thrombocytopenia in some patients.

Liver Function Tests: Median levels of ALT (47.5 U/L) and AST (51.2 U/L) were elevated, corroborating the observed hepatomegaly and indicating EBV's impact on hepatic function.

Biochemical Panel: The panel also measured total bilirubin (TBiL) and lactate dehydrogenase (LDH), which are critical for assessing liver damage and cellular turnover, respectively.

#### 3.1.3 Immunological parameters

Humoral and Cellular Immunity: Immunoglobulin levels (IgM, IgG, and IgA) and T‐cell subsets (CD3 + , CD4 + , CD8 + , CD4 + /CD8 + , CD19 + , CD16 + CD56+ and CD19 + CD23 + ) were quantified. Elevated immunoglobulin levels provide crucial insights into the immune dynamics, reflecting an active immune response to EBV infection.

The baseline characteristics and clinical manifestations elucidated in this study lay the groundwork for understanding the specific impacts of EBV in pediatric populations. Notable trends, such as the high prevalence of fever and organomegaly, are vital for effective diagnosis and management of EBV infections. Moreover, the comprehensive analysis of hematological and immunological parameters offers a detailed snapshot of the infection's clinical profile, enabling the development of targeted therapeutic strategies.

### 3.2 Binary classification of EBV‐DNA copy numbers in plasma and correlation with clinical indicators

This study further analyzed the association between EBV‐DNA copy numbers in plasma and various clinical indicators using a binary classification approach. The cohort was divided based on the median EBV‐DNA copy numbers in plasma into two groups: high EBV‐DNA copy numbers in plasma (>38.9 × 10^2^ copies/ml) and low EBV‐DNA copy numbers in plasma (≤38.9 × 10^2^ copies/ml). This classification facilitated a detailed comparison of clinical and laboratory characteristics between the groups.

#### 3.2.1 Clinical and laboratory findings

Fever Duration and Intensity: There were significant differences in the duration of fever between the groups, with the high EBV‐DNA copy numbers in plasma group experiencing longer fever durations (*p* = .0022), suggestive of a more severe or protracted infection phase.

Hematological Indicators: The platelet count was notably lower in the high EBV‐DNA copy numbers in plasma group (*p* = .0212), indicating a possible aggravation of thrombocytopenic conditions in patients with higher EBV loads.

Liver Function: ALT levels were significantly elevated in the high EBV‐DNA copy numbers in plasma group (*p* = .001), aligning with increased hepatic involvement or damage associated with higher viral loads.

Immunological Response: Significant differences were observed in immunoglobulin levels (IgM, *p* = .0039; IgG, *p* = .0195), pointing towards a heightened immune response or altered B‐cell dynamics in patients with higher EBV‐DNA copy numbers in plasma.

The binary classification revealed critical variances in clinical outcomes related to EBV‐DNA copy numbers in plasma. Notable correlations included:

Inflammatory Markers: Increased lactate dehydrogenase (LDH) levels in the high EBV‐DNA copy numbers in plasma group (*p* = *0.0001*) were indicative of higher cell turnover or tissue damage.

Bilirubin Levels: Total bilirubin (TBiL) was also significantly higher in patients with elevated EBV‐DNA copy numbers in plasma (*p* = .025), suggesting enhanced bilirubin metabolism or liver dysfunction associated with active infection.

Table 2 displays the findings that reveal notable variations in various clinical parameters between the two groups of children infected with EBV, These findings underscore the potential of EBV‐DNA copy numbers in plasma as a biomarker for assessing the severity of infection and guiding clinical management. The differences in clinical and immunological indicators between the two groups highlight the impact of viral load on disease manifestations and severity. The association of higher EBV‐DNA copy numbers in plasma with more severe clinical features and laboratory abnormalities provides evidence for predicting patient outcomes and tailoring therapeutic interventions.

**Table 2:** Binary Classification Analysis of EBV‐DNA copy numbers in plasma and Clinical Indicators.

Characteristic Variable	EBV‐DNA copy numbers in plasma (×10^2^) >38.9*	EBV‐DNA copy numbers in plasma (×10^2^) ≤38.9	*χ2 or t value*	*P value*
Sex (Male/Female)	47/34	44/38	0.1628	0.6866
Age (years)	5.0117 ± 3.0606	4.6443 ± 2.6592	−0.8176	0.4148
Fever (No/Yes)	1/80	6/76	2.3374	0.1263
Peak Fever (°C)	38.6827 ± 4.3909	36.1622 ± 10.2404	−2.0465	**0.0431**
Fever Days	8.0494 ± 4.4614	5.8598 ± 4.5279	−3.1099	**0.0022**
Tonsil Enlargement (No/I/II)	7/64/10	8/66/8	0.3135	0.8549
Cervical Lymphadenopathy (No/Yes)	6/75	8/74	0.0653	0.7983
Hepatomegaly (No/Yes)	39/42	41/41	0.0064	0.9364
Splenomegaly (No/Yes)	39/42	45/37	0.494	0.4821
Eyelid Edema (No/Yes)	44/37	45/37	0	1.0000
Rash (No/Yes)	75/6	73/9	0.2673	0.6051
White Blood Cells (×10^9^/L)	15.522 ± 8.489	14.4772 ± 7.2959	−0.8422	0.4009
Neutrophil%	24.7716 ± 14.1258	26.1356 ± 11.6721	0.6716	0.5028
Lymphocyte%	65.1012 ± 14.0556	64.9268 ± 12.0193	−0.0851	0.9323
Absolute Lymphocyte Count (×10^9^/L)	10.5014 ± 6.8133	9.7624 ± 6.2185	−0.7229	0.4708
Platelets (×10^9^/L)	200.5679 ± 74.4248	227.8293 ± 75.0964	2.3278	**0.0212**
Hemoglobin (g/L)	116.8025 ± 11.3075	118.5 ± 8.0158	1.1045	0.2712
C‐reactive Protein (mg/L)	9.4673 ± 9.2908	12.0982 ± 21.39	1.0206	0.3097
ALT (U/L)	117.8496 ± 125.0293	62.9878 ± 76.5901	−3.3731	**0.0010**
AST (U/L)	88.6086 ± 67.3061	61.5793 ± 58.0377	−2.7443	**0.0068**
lgM	2.0473 ± 0.8175	1.6793 ± 0.7872	−2.9271	**0.0039**
lgG	12.7698 ± 3.6386	11.5095 ± 3.1621	−2.359	**0.0195**
lgA	1.8784 ± 0.8807	1.612 ± 0.8442	−1.9714	0.0504
TBiL (umol/L)	5.8627 ± 3.3938	4.8932 ± 1.8157	−2.2701	**0.0250**
LDH (U/L)	609.1802 ± 175.2267	511.9927 ± 125.7132	−4.0643	**0.0001**
CD3+ Absolute Count(×10^9^/L)	8.5438 ± 5.6023	7.8699 ± 4.8583	−0.8200	0.4134
CD4+ Absolute Count(×10^9^/L)	1.6842 ± 1.443	1.8124 ± 1.666	0.5254	0.6001
CD8+ Absolute Count(×10^9^/L)	6.0696 ± 4.0998	5.5387 ± 3.3746	−0.9021	0.3684
CD4 + /CD8+	0.3901 ± 0.5437	0.4 ± 0.3095	0.1423	0.8871
CD19+ Absolute Count(×10^9^/L)	0.6451 ± 0.613	0.7239 ± 0.7117	0.7579	0.4496
CD(16 + 56)+ Absolute Count(×10^9^/L)	1.1684 ± 1.3989	1.0257 ± 1.0833	−0.7276	0.4680
CD19 + CD23+ Absolute Count(×10^9^/L)	0.3268 ± 0.3426	0.3689 ± 0.3626	0.7611	0.4477
Blood Smear Neutrophil%	26.963 ± 14.821	26.4024 ± 12.7123	−0.259	0.7960
Blood Smear Lymphocyte%	61.6049 ± 14.0131	63.4146 ± 11.7819	0.8919	0.3738
Blood Smear Atypical Lymphocyte Ratio%	5.0247 ± 6.4342	4.1951 ± 4.8367	−0.9296	0.3541
Ferritin (ug/L)	223.5556 ± 374.2909	124.5744 ± 106.2785	−2.2906	**0.0243**
Hospital Days	10.3704 ± 3.4112	8.7317 ± 3.251	−3.1388	**0.0020**

* Optimal cut‐off value: 38.9.

### 3.3 Binary classification of hospital days and correlation with clinical indicators

In this analysis, the study cohort was further divided based on the median hospital stay into two groups: longer hospital stays (>9 days) and shorter hospital stays (≤9 days). This stratification allowed for an examination of the association between the length of hospitalization and various clinical and laboratory parameters, potentially indicative of more severe disease states or complications.

#### 3.3.1 Key clinical and laboratory variations

Tonsil Enlargement and Inflammation: Significant differences were observed in tonsil enlargement (*p* = .0024), with the longer stay group showing a higher prevalence, suggesting more severe oropharyngeal involvement.

Hematological Parameters: A lower platelet count was noted in the group with longer hospital stays (*p* = .0045), which may be reflective of prolonged disease activity or secondary complications affecting bone marrow function.

#### 3.3.2 Immune response and organ function

LDH Levels: Elevated LDH levels in the longer stay group (*p* = .0394) indicated increased cellular turnover, which could be associated with more extensive tissue damage or a higher degree of inflammatory response.

#### 3.3.3 Statistical insights and clinical implications

Binary Classification Analysis: The findings from the binary classification according to hospital stay duration revealed noteworthy differences in several key clinical indicators, directly correlating with the intensity and progression of EBV‐associated symptoms and complications.

Inflammatory and Immune Markers: Ferritin levels, an acute phase reactant, were significantly higher in the longer stay group (*p* = .0106), reflecting a more intense inflammatory response or ongoing infection.

EBV‐DNA copy numbers in plasma: Furthermore, a significant difference in EBV‐DNA copy numbers in plasma (*p* = *0.0361*) between the groups highlights the potential of viral load as a predictor of disease severity and the necessity for prolonged hospitalization.

Table 3 displays notable variances in the duration of hospital stay in pediatric EBV infections is closely linked with several clinical and laboratory parameters, indicating its utility in assessing disease severity. The associations observed support the use of these parameters in clinical practice to identify patients at risk of severe outcomes, thereby facilitating timely and targeted interventions.

**Table 3:** Binary Classification of Hospital Days and Correlation with Clinical Indicators.

Characteristic Variable	Hospital Days > 9*	Hospital Days ≤ 9	*χ2 or t value*	*P value*
Sex (Male/Female)	37/32	54/40	0.1063	0.7444
Age (years)	4.6932 ± 2.7718	4.925 ± 2.9386	0.5140	0.6080
Fever (No/Yes)	2/67	5/89	0.1312	0.7172
Peak Fever (°C)	37.9928 ± 6.637	36.9904 ± 8.8354	−0.8270	0.4094
Fever Days	7.2174 ± 4.8662	6.75 ± 4.4352	−0.6288	0.5305
Tonsil Enlargement (No/I/II)	8/47/14	7/83/4	12.0403	**0.0024**
Cervical Lymphadenopathy (No/Yes)	6/63	8/86	<0.001	1
Hepatomegaly (No/Yes)	40/29	40/54	3.1930	0.0740
Splenomegaly (No/Yes)	37/32	47/47	0.0892	0.7652
Eyelid Edema (No/Yes)	41/28	48/46	0.8092	0.3684
Rash (No/Yes)	64/5	84/10	0.2171	0.6412
White Blood Cells (×10^9^/L)	15.2438 ± 8.6028	14.8148 ± 7.3917	−0.3336	0.7392
Neutrophil%	25.2684 ± 14.1195	25.5968 ± 12.0543	0.1559	0.8763
Lymphocyte%	64.4029 ± 14.3047	65.4617 ± 12.0699	0.4983	0.6191
Absolute Lymphocyte Count (×10^9^/L)	10.2897 ± 6.9932	10.0121 ± 6.1693	−0.2630	0.7929
Platelets (×10^9^/L)	196.2174 ± 72.313	230.734 ± 79.629	2.8840	**0.0045**
Hemoglobin (g/L)	118.3768 ± 10.1098	117.1277 ± 9.5818	−0.7968	0.4269
C‐reactive Protein (mg/L)	10.0367 ± 12.3317	11.3444 ± 19.075	0.5306	0.5965
ALT (U/L)	88.3872 ± 112.784	91.6181 ± 102.8094	0.1875	0.8515
AST (U/L)	75.2435 ± 66.84	74.8404 ± 62.3221	−0.0391	0.9688
lgM	1.991 ± 0.9088	1.7676 ± 0.7407	−1.6746	0.0964
lgG	12.2628 ± 3.1761	12.0426 ± 3.6601	−0.4098	0.6825
lgA	1.6961 ± 0.7589	1.7798 ± 0.946	0.6262	0.5321
TBiL (umol/L)	5.8046 ± 3.4941	5.0596 ± 2.0067	−1.5893	0.1151
LDH (U/L)	592.5826 ± 197.9916	536.583 ± 119.6282	−2.0864	**0.0394**
CD3+ Absolute Count(×10^9^/L)	8.069 ± 5.4491	8.3045 ± 5.101	0.2801	0.7798
CD4+ Absolute Count(×10^9^/L)	1.7595 ± 1.5217	1.7408 ± 1.5882	−0.0762	0.9394
CD8+ Absolute Count(×10^9^/L)	5.6589 ± 3.972	5.908 ± 3.5969	0.4115	0.6814
CD4 + /CD8+	0.4754 ± 0.6008	0.3362 ± 0.256	−1.8076	0.0742
CD19+ Absolute Count(×10^9^/L)	0.7156 ± 0.7275	0.662 ± 0.6156	−0.4950	0.6214
CD(16 + 56)+ Absolute Count(×10^9^/L)	1.3277 ± 1.6106	0.927 ± 0.8654	−1.8770	0.0635
CD19 + CD23+ Absolute Count(×10^9^/L)	0.3583 ± 0.381	0.3404 ± 0.3316	−0.3129	0.7549
Blood Smear Neutrophil%	27.6957 ± 14.4866	25.9362 ± 13.2314	−0.7945	0.4282
Blood Smear Lymphocyte%	61.1159 ± 13.5988	63.5426 ± 12.3903	1.1684	0.2446
Blood Smear Atypical Lymphocyte Ratio%	4.3768 ± 5.8337	4.7766 ± 5.5979	0.4397	0.6608
Ferritin (ug/L)	248.9638 ± 407.0622	118.5596 ± 78.7938	−2.6252	**0.0106**
EBV‐DNA copy numbers in plasma (×10^2^copies/ml)	469.577 ± 1410.8917	102.6229 ± 249.54	−2.1361	**0.0361**

* Optimal cut‐off value: 9.

## 4 DISCUSSION

Similar to other herpesviruses, EBV invades epithelial cells and circulating B lymphocytes following initial infection, remaining latent in the body for prolonged periods.^11^ If the balance between the host and the virus is disturbed, the virus may reactivate. Various diseases are linked to primary or reactive EBV infections, including infectious mononucleosis (IM), respiratory infections, encephalitis, malignant lymphomas, nasopharyngeal carcinoma, aplastic anemia, hemophagocytic lymphohistiocytosis (HLH), immunodeficiency, and autoimmune diseases.^12^

Diagnosing EBV infection primarily depends on serological tests and molecular biology methods. However, the accuracy of serological tests may be compromised by the underdeveloped immune system in children and the presence of maternal antibodies in infants, leading to false‐negative or false‐positive results.^13^ Molecular biology testing has become an essential tool for diagnosing and monitoring EBV infections and associated diseases in immunocompromised hosts.^14^ Prior research indicates^15^ that serological and molecular biology tests should be used complementarily to diagnose a patient's EBV infection status.^16^

In our study, We grouped these patients based on EBV copy numbers and hospital stay durations, compared clinical indicators across groups, and clarified the correlation between various laboratory indicators and clinical indicators to improve the level of diagnosis and treatment. Our results indicate significant differences in fever duration, platelet count, ALT, immunoglobulin IgM, IgG, TBiL, LDH, and hospital stay duration based on EBV‐DNA copy numbers in plasma classifications. This suggests that EBV‐DNA copy numbers in plasma might correlate with these clinical variables. Additionally, the classification based on hospital days revealed significant differences in tonsil enlargement, platelet count, LDH, ferritin, and EBV‐DNA copy numbers in plasma, suggesting a potential correlation between hospital stay duration and these clinical variables.

Children in the high copy number group also had higher ALT and TBiL levels, indicating that higher EBV‐DNA copy numbers in plasma are more likely to cause liver damage, consistent with findings by Lin Shengjing.^17^ Therefore, children in the high copy number group require more active monitoring of liver function. Fever is the most common clinical manifestation in children with EBV infections, and the duration of fever often varies. Some children with EBV infections need hospital treatment due to their condition, and due to individual differences in disease progression, the duration of hospital stays also varies. Our study found that children in the high copy number group had longer fever and hospital stay durations. Thus, EBV‐DNA copy numbers in plasma can be an important indicator for assessing the duration of fever and clinical outcomes in children. However, this indicator is limited by factors such as the inability to perform and long turnaround times for results in some primary care hospitals.

Our findings also showed that platelet count and LDH correlate with EBV‐DNA copy numbers in plasma, suggesting that these indicators can be used to assess the duration of fever and clinical outcomes in children. To further validate this observation, our study classified hospital stay durations based on the median value, and the analysis yielded similar results. Moreover, these indicators are more readily available and have higher clinical testing efficiency in primary care hospitals, thus holding greater clinical value.

Our findings demonstrate that EBV viral load and the subsequent length of hospital stay are correlated with clinical indicators. Platelets, small anucleate cells circulating in the blood for about 7 to 10 days, primarily function in hemostasis by forming clots to protect the integrity of blood vessels. They originate from megakaryocytes, which are large polyploid cells found in the bone marrow and are developed from hematopoietic stem cells. As megakaryocytes mature internally, they develop proplatelets that release into the bloodstream.^18^ Platelets typically circulate at levels between 150 and 450×10^9^/L when bone marrow is functioning normally.^19^ Different cytokines, such as stromal cell‐derived factor 1 (SDF‐1), granulocyte‐macrophage colony‐stimulating factor (GM‐CSF), interleukins (IL‐3, IL‐6, and IL‐11), fibroblast growth factor 4 (FGF‐4), and thrombopoietin (TPO), play a role in stimulating the production of megakaryocytes, with TPO being the most important.^20^ While TPO plays a vital role in supporting hematopoietic stem cells,^21^ the majority of these cytokines have pro‐inflammatory properties and can prompt the quick maturation and activation of white blood cells, leading to an increase in megakaryocyte production. This paper elucidates the impact of inflammatory processes on platelet production.

Previous research suggested that acute EBV infections could lead to thrombocytopenia in children.^22^ A recent study by Zhang et al.^23^ Reported a rare case involving a 14‐year‐old girl experiencing severe thrombocytopenia, with a platelet count dropping to 5×10^9^/L, and showing signs of spontaneous bleeding along with periorbital edema, which is an uncommon symptom of EBV‐related infectious mononucleosis. After receiving intravenous immunoglobulin and a 4‐day regimen of methylprednisolone, leading to her discharge 7 days after being admitted a platelet count of 143×10^9^/L. It is important to keep in mind the possibility of EBV infection when diagnosing acute severe thrombocytopenia.

Lactate accumulation typically results in a lactate (LA) microenvironment, often associated with worse outcomes in cancer progression, metastasis, and decreased disease‐free and overall survival.^24^ Lactate dehydrogenase (LDH) is found in many different tissues and can be detected in the blood because it helps convert pyruvate to lactate, a reaction that is rapid and near‐equilibrium, highly dependent on local lactate gradients.^25^ An increase in LDH and lactate production is considered a poor prognostic aspect in several malignancies including solid tumors,^26^ but the mechanisms by which oncogenic viruses exploit this metabolic process for cancer progression and adaptation to acidic conditions are still not well understood. Evidence indicates that dynamic interactions between EBV and surrounding environmental factors, such as hypoxic stress, is closely linked to the aggressive behavior of host cells. EBV has the ability to stimulate anaerobic glycolysis in NPC and B‐cell lymphomas by upregulating the oncogenic proteins LMP1 and HIF‐1α.^27^ Our findings support that LDH could be a significant marker reflecting EBV viral effects.

LDH is an enzyme widely present in various tissue cells of the human body. When cells are damaged or undergo necrosis, LDH is released from within the cells into the bloodstream, reflecting the metabolic state and the extent of tissue damage.^28^ As an intracellular enzyme, LDH is released into the blood when cells are damaged or necrotic, leading to elevated serum LDH levels, which indicate the degree of cellular necrosis and the immune status of the body. In this study, we grouped these patients based on EBV copy numbers and hospitalization duration. The results showed statistically significant differences in LDH levels among the groups, indicating that higher plasma EBV DNA copy numbers are more likely to induce immune‐inflammatory responses and inflammatory damage, resulting in prolonged hospitalization in affected children.

This study is primarily limited by its retrospective design and dependence on data from a single center, which may contribute to selection bias and limited regional representativeness. Future studies might consider more detailed subgroup analyses based on age, sex, and severity of clinical manifestations to determine these factors’ specific effects on the clinical progression of EBV infections. A prospective cohort design and expansion to multiple centers would help reduce information bias in retrospective studies, enhance the generalizability of the research, and improve the reliability of the conclusions. Additionally, controlling for more potential confounders and increasing the sample size are recommended.

## 5 CONCLUSION

Infectious mononucleosis, caused by EBV infection, is a common disease in children with varying degrees of severity. Some children require hospitalization, and those with longer hospital stays have higher LDH levels compared to those with shorter stays. Our study confirms that changes in platelet counts and lactate dehydrogenase levels may serve as important indicators of clinical outcomes in children with EBV infections, providing new monitoring markers for clinical treatment.

## AUTHOR CONTRIBUTIONS

**Yan Li**: Conceptualization; Data curation; Investigation; Methodology; Project administration; Writing—original draft. **Kun Wang**: Data curation; Funding acquisition; Validation; Visualization; Writing—review and editing.

## CONFLICT OF INTEREST STATEMENT

The authors have no conflict of interest.

## ETHICS STATEMENT

This study was approved by Children's Hospital of Soochow University ethics committee (2023CS222). Due to the retrospective nature of the study, patient consent was not required.

## Supporting information

Supporting information.

## Data Availability

All raw data and code are available upon request.
